# Are There Mutator Polymerases?

**DOI:** 10.1100/tsw.2003.32

**Published:** 2003-05-27

**Authors:** Miguel Garcia-Diaz, Jose F. Ruiz, Raquel Juarez, Gloria Terrados, Luis Blanco

**Affiliations:** Centro de Biología Molecular Severo Ochoa (CSIC-UAM). Universidad Autónoma, 28049 Madrid, Spain

**Keywords:** DNA polymerase, mutagenesis, DNA repair, tranlesion synthesis, somatic hypermutation, VDJ recombination

## Abstract

DNA polymerases are involved in different cellular events, including genome replication and DNA repair. In the last few years, a large number of novel DNA polymerases have been discovered, and the biochemical analysis of their properties has revealed a long list of intriguing features. Some of these polymerases have a very low fidelity and have been suggested to play mutator roles in different processes, like translesion synthesis or somatic hypermutation. The current view of these processes is reviewed, and the current understanding of DNA polymerases and their role as mutator enzymes is discussed.

## INTRODUCTION

The last 3 years have seen a revolution in the field of DNA replication and repair; unchanged for the last years, the list of identified DNA polymerases has expanded to more than twice its original number. Along with the discovery of novel members of long-known families and the appearance of a new family of enzymes (family Y[1]), the outcome of this burst of discovery has been a strongly modified concept of what a DNA polymerase is, and what it does in the cell.

Until recently, DNA polymerases have been generally conceived as extremely precise enzymes whose main cellular function is to faithfully replicate the genome. This point of view arose from the study of enzymes as *E. coli* Pol III, or eukaryotic Pols ε and δ, that are extremely processive (i.e., able to polymerize a large number of nucleotides without dissociating from the template) and endowed with an accessory 3’-5’ exonucleolytic proofreading, that guarantees a high fidelity of synthesis. Under this assumption, the introduction of an incorrect dNMP into DNA is a rare and fortuitous event, and mutations arise mainly as a consequence of a defect in the replication process.

However, the biochemical analysis of the recently discovered polymerases has proven to be extremely shocking; most of them have an unprecedented error-proneness, that raises the question of their possible function into the cell. While a few have been related to DNA repair, some are starting to be considered as mutagenic, and different roles related to the generation of variability in the genome are being proposed (Table 1).

*Table I.- DNA polymerases proposed to participate in mutagenesis*

| Name | Organism | Year of discovery | Family | Proposed functions |
|---|---|---|---|---|
| TdT | Eukaryotes | 1960 | X | V(D)J recombination |
| Pol ζ | Eukaryotes | 1996 | B | Mismatch extension<br>Somatic hypermutation<br>Translesion synthesis |
| Pol η | Eukaryotes | 1999 | Y | Translesion synthesis<br>Somatic hypermutation |
| Pol IV | *E. coli* | 1999 | Y | SOS response<br>Translesions synthesis<br>Spontaneous mutagenesis |
| Pol V | *E. coli* | 1999 | Y | SOS response<br>Translesions synthesis |
| Pol μ | Eukaryotes | 2000 | X | Somatic hypermutation<br>NHEJ |
| Pol ι | Eukaryotes | 2000 | Y | Translesion synthesis<br>Somatic hypermutation<br>Base excision repair |
| Pol κ | Eukaryotes | 2000 | Y | Translesion synthesis |

## V(D)J RECOMBINATION

Paradoxically, one of the first eukaryotic DNA polymerases to ever be identified[2] was mammalian terminal deoxynucleotidyl transferase (TdT), a family X enzyme with polymerization properties largely different to what would be expected for a replicative polymerase; its preferred substrate is an untemplated DNA. After its first characterizations[3,4,5], TdT has not been extensively studied as has been the case for the model enzyme of its family, Pol β[6]. However, the interest for the biochemical properties of this enzyme has risen recently[7,8,9], and this year Delarue et al. have reported a three-dimensional structure of the enzyme complexed to DNA that has allowed the authors to suggest a structural basis for its preference for an untemplated substrate[10]. Far from being involved in the replication process and in agreement with its properties, the *in vivo* function of TdT appears to be related to the impressive variability associated to immunoglobulin gene chain rearrangements.

Specific recognition of antigens by lymphocytes is achieved through the action of heterodimeric protein receptors known as immunoglobulins (Igs) and T cell receptors (TCR), for B and T lymphocytes, respectively. In order to cope with an almost infinite number of different possible antigens, lymphocites undergo a number of variability-generating processes that allow a somatic diversification of Ig and TCR genes. Starting with a limited set of germline sequences, all these processes allow humans to generate a variability of about $10^{14}$ and $10^{18}$ different clones for

Igs and TCRs, respectively[11]. In vertebrates, three such processes are known: V(D)J recombination[12,13], somatic hypermutation[14,15], and class-switching recombination[16].

V(D)J recombination takes place exclusively in lymphocyte precursors in thymus and bone marrow. At a specific stage of lymphocyte development, a mature Ig or TCR gene is assembled out of a series of germline gene segments through a process of specific recombination. However, recombination by itself does not account for the whole diversifying potential of this process, which is mainly guaranteed by the action of a nonorthodox DNA polymerase: TdT. Indeed, making use of its peculiar properties, TdT inserts untemplated nucleotides at the junctions of the rearranging segments, thus generating a broad spectrum of mutations that allow a greater diversity of the antigen receptor "repertoire".

## SOMATIC HYPERMUTATION

Further diversification of antigen receptor specificity takes place in mature B lymphocytes. Upon antigen recognition, B lymphocytes migrate to secondary lymphoid tissues, where they undergo a process of clonal expansion and affinity maturation in structures called germinal centers. This affinity maturation step is called somatic hypermutation (SH), and involves the highly frequent mutation of the variable regions of B lymphocites Ig genes. The mechanism by which mutations are introduced is yet unknown, but it has been suggested that a DNA polymerase with mutagenic properties could be the "mutator" enzyme responsible for Ig gene variability[17]. Indeed, recent evidence suggests that SH is triggered by a process of site-directed DNA cleavage that would generate DNA breaks or nicks throughout the variable regions of the Ig gene. These nicks or breaks would thus provide the 3'-OH ends required for DNA polymerase action. Another fact that supports the existence of a "mutator" enzyme is the astonishingly high mutation frequency, which is otherwise difficult to explain even in the total absence of DNA repair.

The first proposal of a candidate DNA polymerase was made 2 years ago by Domínguez et al.[18], and later supported by other groups[19]. This proposal was based on the observed biochemical properties and expression profile of a novel DNA polymerase from family X, DNA polymerase mu (Pol μ). This enzyme, which is closely related to TdT (42% amino acid identity), also displays a template-independent polymerization capacity, although it largely prefers a templated substrate[18]. However, the main fact that points out Pol μ as a candidate "hypermutator" is its extremely high infidelity[20,21], together with its expression pattern, that is predominant in secondary lymphoid organs.

Shortly after the discovery of Pol μ, Tippin and Goodman[22] proposed a family Y enzyme, pol ι, as a candidate to participate in SH. This hypothesis was originally based on the reduced fidelity of this enzyme[23], and was further supported with the observation that the fidelity of this enzyme is lowered at the end of DNA templates, suggested to be the substrate of SH[24].

Shortly after this proposal, another candidate polymerase from family Y emerged. Analysis of Xeroderma pigmentosum patients (a deficiency in the repair of UV-damage in DNA) deficient in the low fidelity Pol η showed an altered spectra of SH-generated mutations compared to normal individuals[25], and the comparison of the *in vitro* mutation specificity of Pol η with the mutational spectra of different Ig genes of healthy individuals suggested that this polymerase could play an important role in SH[26].

The list of candidate DNA polymerases was lastly completed when a recent study proposed that Pol ζ, a DNA polymerase with a high mispair extension capacity, might be also essential for somatic hypermutation[27].

## TRANSLESION SYNTHESIS

Apart from their proposed role in secondary lymphoid response, Pols η and ι are known to have a more general role in the cell: the copying of replication-stalling DNA lesions (such as generated by different mutagens as UV light) in a process known as "translesion synthesis" (TLS[28,29,30,31]). Most polymerases, including those responsible for chromosome replication, are unable to replicate past these bulky DNA lesions, whose persistence in the DNA would cause replication to stop. TLS polymerases, however, are able to polymerize past those lesions, allowing the replication machinery to circumvent them.

In eukaryotes, four polymerases are currently believed to participate in the TLS process: three TLS polymerases (family Y Pols η, κ and ι), that efficiently catalyze synthesis opposite different DNA lesions *in vitro*[32,33,34,35,36,37], and a mispair extender (family B Pol ζ) that, although unable to polymerize opposite lesions, might be sometimes required to extend past the site of repair following the action of a TLS polymerase[38]. Moreover, a similar mispair extension capacity has recently been reported for Pol κ, thus raising the possibility that both Pol κ and ζ might alternatively contribute to the extension activity of TLS[39].

The major drawback of the TLS process is that replication past DNA lesions is not costless; the damaged nucleotides are frequently miscoding, and thus bypass is achieved at the cost of introducing mutations into the genome. Why TLS repair is sometimes preferred over error-free forms of repair (such as recombinational repair) is unknown, although it is believed that TSL might be limited to situations in which the accumulation of DNA damage exceeds the repair capacity of error-free pathways, or to situations in which a DNA lesion is encountered by the replication fork.

However, TLS might not always be an error-prone process. Surprisingly, although being an extremely unfaithful enzyme when copying undamaged templates[40,41], DNA polymerase η is able to bypass some DNA lesions in an error-free manner[42]. Indeed, lack of Pol η activity in humans leads to an increased mutagenesis in response to UV light[43], probably because lesion bypass is then carried out by an error-prone polymerase.

Still, whether a particular TLS polymerase is error-free or error-prone probably depends on the nature of the substrate DNA lesion; each of the TLS polymerases appear to have specificity for a particular lesion[36,44,45,46,47,48,49], and it is likely that other TLS polymerases might compete with the preferred (error-free) polymerase for a specific substrate. For instance, synthesis by Pol η can also have mutagenic consequences in some DNA lesions[50,51], and pol η-expressing yeast cells show increases in their spontaneous mutagenesis rate[52]. Similarly, Pols κ and ι show error-free and error-prone activities *in vitro* on different substrates.

## TRANSLESION SYNTHESIS IN BACTERIA: THE SOS RESPONSE

The SOS-induced repair pathway has been known for decades to cause the accumulation of mutations in *E. coli* in response to DNA damage[53]. Although the mechanism by which these mutations are introduced was unknown, the existence of low-fidelity DNA polymerases was already considered as a likely possibility more than 20 years ago[54]. Today, the molecular characteristics of SOS-repair are better understood. This pathway is responsible for the induction of a number of genes upon DNA damage[55], among which are three *E.coli* DNA polymerases: DNA polymerases II, IV, and V. Although the exact roles of each of these polymerases are still not completely elucidated, it seems clear that Pol IV and especially Pol V can be directly accounted for the mutagenic consequences of repair; both polymerases carry out error-prone TLS[56,57,58]. However, the fact that in bacteria TLS poorly contributes to DNA damage resistance[59], has led to the hypothesis that the function of the SOS pathway might be the generation of mutations that would allow the adaptation of a bacterial population to genotoxic conditions. Moreover, it is known that *E. coli* undergoes a process of uninduced or spontaneous mutagenesis, of unclear biological significance, and there is evidence that suggests the involvement of Pol IV in this process[60,61,62].

## STRUCTURAL BASIS OF INFIDELITY FOR FAMILY Y POLYMERASES

Recently, a number of crystal structures of family Y DNA polymerases has provided an explanation for the extreme infidelity of these enzymes, and their ability to replicate bulky DNA lesions[63,64,66]. Shown in Fig. 1A is the crystal structure of Dpo4 from *Sulfolobus solfataricus* complexed with a DNA template/primer[64]. In contrast with other known polymerase structures, Dpo4 presents a small fingers domain, resulting in an extremely open nascent basepair binding pocket, that allows to accommodate bulky lesions in the catalytic site, a capacity that is crucial for TLS. However, the active site of Dpo4 can accommodate not only DNA lesions, but also (as can be seen in Fig. 1B and C) template misalignments. This feature is probably the basis for the reduced fidelity of this enzyme[67]. Thus, the intrinsic error-proneness of family Y enzymes on undamaged DNA templates might be the unwanted (?) side-effect of their ability to replicate bulky lesions.

## OTHER NOVEL POLYMERASES FROM FAMILY X

Besides Pol $\mu$, already discussed in this paper, the family X of DNA polymerases has grown by two other new members: Pol $\lambda$[68,69,70] and Pol $\sigma$[71]. Although its cellular role is unknown, Pol $\lambda$ seems to be present in all eukaryotes, including plants[68], and is expressed in all tissues examined, suggesting a general role for this enzyme. The fact that it has a dRP lyase activity[72] (an activity crucial for the Base Excision Repair pathway) together with its biochemical characteristics[73] suggest that Pol $\lambda$ is involved in DNA repair. Its base substitution fidelity seems to be relatively high (similar to that of Pol $\beta$), but further characterization of the fidelity of this enzyme is required to evaluate its potential role in mutagenesis.

Pol $\sigma$ has been exclusively characterized in yeast, although it is believed to be present in other eukaryotes as well[74]. Its main function seems to be related to sister chromatin cohesion[71], but little is known about the biochemical characteristics of this enzyme, that remain controversial[75], or about its fidelity of synthesis.

## DISCUSSION

V(D)J recombination, SH, and TLS are three cellular processes for which the need for a mutator polymerase has been invoked. Both V(D)J recombination and SH are clear examples of mutagenic processes that are triggered by the cell itself. However, whereas the role of TdT in V(D)J recombination has been clearly established, the mutagenic mechanism of SH is currently unclear. Although several evidences support the participation of each candidate DNA polymerase, there is evidence suggesting that these proposals should be carefully reconsidered. Thus, and despite the fact that studies with Pol $\iota$ deficient cell lines seem to suggest the involvement of this polymerase in the SH process[76], the analysis of the pattern of SH mutations[77], the analysis of human deficiencies (Pol $\eta$[25]), and the generation of mice ko models (Pol $\mu$[78], Pol $\kappa$[79]) have failed to prove the involvement of any particular enzyme. Instead, these data suggest that SH might depend on the action of more than one DNA polymerase, and therefore a single enzyme might be dispensable for the process. The answer to this possibility will be found in the generation of ko mice deficient in multiple DNA polymerases.

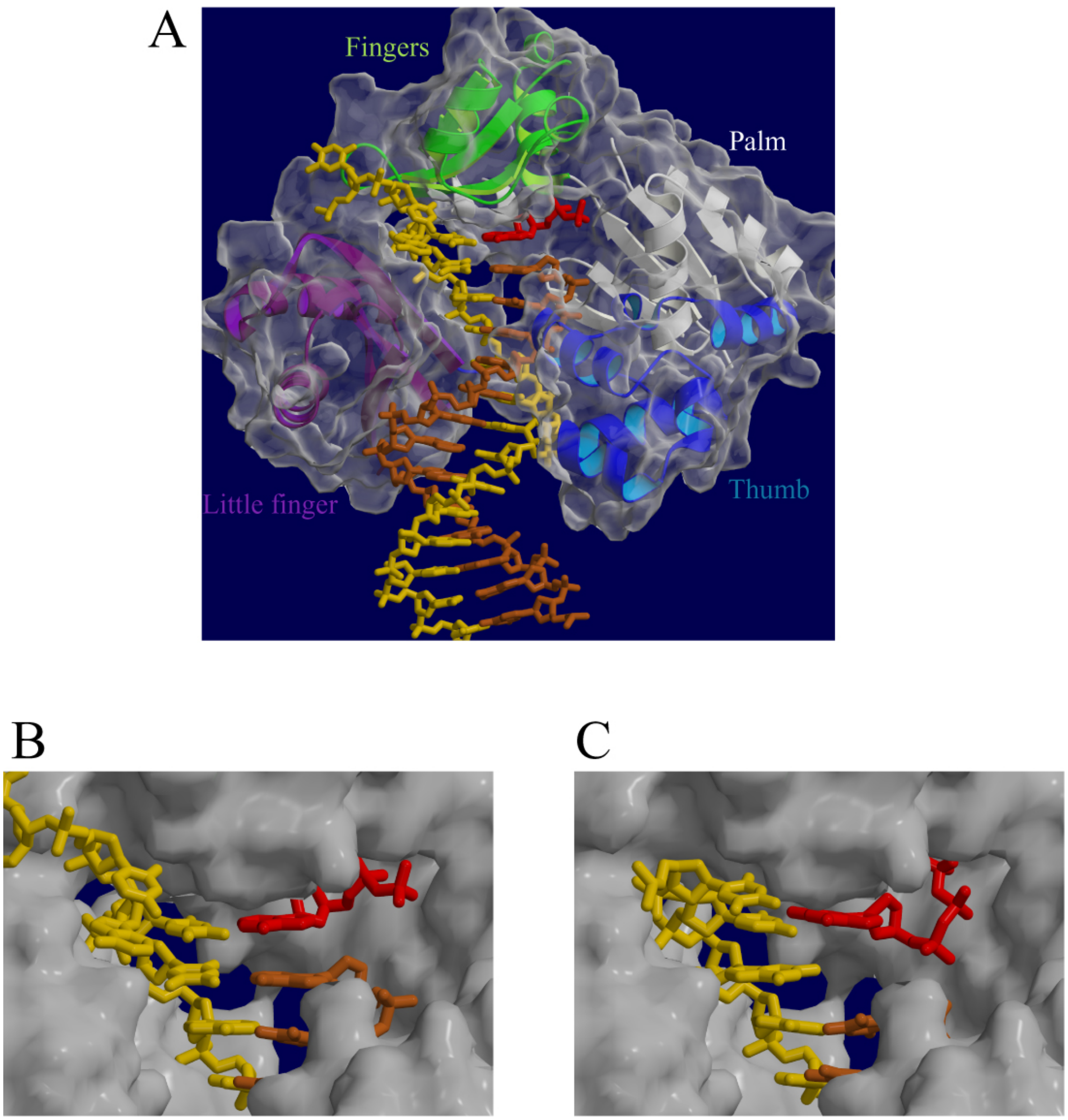

**FIGURE 1.** A: Ternary complex of *Sulfolobus sulfataricus* Dpo4 complexed with a DNA template-primer (yellow and brown, respectively) and an incoming nucleotide (red). A transparent molecular surface representation of the enzyme shows that the fingers and thumb domains are exceptionally small when compared to other DNA polymerases, thus configuring an extremely open nascent basepair binding pocket[64]. The nascent basepair binding pocket is able to accommodate a correct basepair (B) as well as a misaligned template primer (C). The figure was made with Molscript[80] and GRASP[81] and rendered with Raster3D[82].

TLS polymerases, on the other hand, are certainly involved in mutagenesis, at least in bacteria and lower eukaryotes. However, the meaning of such a process is still a matter of debate. Although these polymerases bear mutagenic consequences to the cell, it can not be discarded that the mutations arising as a consequence of TLS are the price to pay for keeping a "safeguard" system able to maintain the delicate equilibrium between mutagenesis and cell viability.

Thus, although it is clear that cells harbor many unfaithful and peculiar enzymes, no conclusions can be drawn yet as to the presence of "mutator" DNA polymerases into the cell, with the exception of the long-known and sometimes forgotten TdT. Whether one or more of the novel enzymes will add to the list of "creative" polymerases, maybe related to a process not yet foreseen, will be one of the most intriguing and exciting questions of the field in the years to come.

## ACKNOWLEDGMENTS

We thank Drs. Joe Krahn and Bill Beard for help in the generation of figures, and Dr. Katarzyna Bebenek for critical reading of the manuscript. This work was supported by DGES (PB97-1192), DGI (BMC2000-1138), and CAM (08.1/0044/98 and 08.5/0063/2000) and by an institutional grant from Fundación Ramón Areces.
